# Supplementary material for: Impingement of systems containing suspending droplets and coming ones at the nanoscale
Source: PLoS One. 2026 Feb 2;21(2):e0340439. doi: 10.1371/journal.pone.0340439 (PMC12863518; doi:10.1371/journal.pone.0340439)
Supplement: Attachment — Submitted filename: Response to Reviewer #3.docx [file pone.0340439.s002.docx]

**Response to Reviewer**

The authors studied the impingment of two separate nanodrops on a solid surface. The work is interesting and falls in the scope of Plos One. The manuscript need to be further improved to be accepted in Plos One.
Ans: First of all, thank you for your comments, which are very helpful in improving our paper. Subsequently, we tried our best to revise our manuscript and addressed your comments point-by-point. Please see the following responses. Please note that the corrections are written in red and the responses are written in blue in both this response letter and the marked-up manuscript.

1. Each abbreviation should be specified when first used, such as MD in the Abstract.

Ans: Thank you for your valuable comment. We have added the full name when the abbreviation is first used in revised manuscript.

2. Each symbol should be specified when first used, such as D_sep and We in the Abstract.

Ans: Thank you for your valuable comment. We have added the full name when each symbol is first used in revised manuscript.

3. Please improve the English of the manuscript. For example, “The representative dynamics under different given conditions.” seems incomplete. Please check the sentence from Line 21 to Line 22 in Page 3.

Ans: Thank you for your valuable comment. We have modified the sentence as “The representative dynamics under different given conditions have been directly captured with the help of MD simulations.”

(Please see Page 1, Lines 12-14.)

4. Please specify the external factor and the prefactor in the Abstract.

Ans: Thank you for your valuable comment. Please see the following statement in the revised manuscript: “The external factor of *D*_sep_ can not affect the primary spreading but shows a significant influence on the secondary spreading. The prefactor of the secondary spreading at low-velocity impingement is 0.06 whose value is the same as that for primary spreading. On further increasing *We*, the high-velocity impingement alters the secondary spreading with prefactor increasing from 0.06 to 0.09.”

(Please see Page 1, Lines 19-22; Page 2, Line 1.)

5. Each symbol should be used for only one thing. For example, epsilon was used to represent both the coefficient of restitution and the depth of the potential wall.
Ans: Thank you for your valuable comment. We apologize for stupid mistakes and rewritten the sentence “The coefficient of restitution, *ε*_co_, can reflect bouncing kinetic characteristics in another way, which is defined as the ratio between bouncing energy to impacting energy.”

(Please see Page 4, Lines 10-12.)

“The *ε*_co_ strictly obeys a law of *We^-^*^1/4^ at low *We* range, while such a law is replaced by *ε*_co_~*We^-^*^2/3^ after increasing the Weber number to a relativey high value.”

(Please see Page 4, Lines 13-15.)

6. Please add unit for (0,0,12) and (0,0,29) in Line 14, Page 6.
Ans: Thank you for your valuable comment. We have rewritten the sentence as “Droplets are completely identical with a diameter of 8 nm, containing 8900 water molecules, and the center-of-mass coordinates of two suspended droplets are (0 nm, 0 nm, 12 nm) and (0 nm, 0 nm, 26 nm), respectively.”

(Please see Page 6, Lines 15-17.)

7. Please specity mW water model in Line 20, Page 6.

Ans: Thank you for your valuable comment. Please see the following statement in the revised manuscript: “We perform a mW (monoatomic water) model to describe the intermolecular interactions between water molecules. Such a model is coarse-grained, which uses a large group for simplifying the structure of water molecules.”

(Please see Page 7, Lines 1-3.)

8. Please check the unit of the viscosity in Line 2, Page 7.

Ans: Thank you for your valuable comment. Please see the following statement in the revised manuscript: “the viscosity of the model is *μ*=283.7μPa.s…”

(Please see Page 7, Lines 6-7.)

9. It is better to include D_sep in Figure 1.

Ans: Thank you for your valuable comment. We have added *D*_sep_ in Figure 1 in revised manuscript.

**Figure 1.** The targeted system of the present work contains two identical nanodroplets with a diameter of 8 nm. Two droplets are put into a vacuum environment with a separated distance of 6 nm.

(Please see Pages 7-8.)

10. “Owing to the existence of a high difference” in Line 18, Page 8 might be “owing to the existence of a height difference”.

Ans: Thank you for your valuable comment. Please see the following statement in the revised manuscript: “owing to the existence of a height difference…”

(Please see Page 9, Line 14.)

11. What does z-direction in Figure 3b mean?

Ans: Thank you for your valuable comment. It means that the position of each two droplets in the *z*- direction.

12. Please specify what a high We range is in Line 17, Page 10.

Ans: Thank you for your valuable comment. Please see the following statement in the revised manuscript: “At high *We* range of *We*>100, …”

(Please see Page 12, Line 3.)

13. Please check whether the sentence “When a number is selected, no matter how D_sep changes” is correctly expressed for the authors only chose three values of D_sep on the order of 10 nm.

Ans: Thank you for your valuable comment. We have rewritten the sentence as “When the Weber number is selected, the primary spreading has a consistent trend of change with reaching the same maximum primary spreading factor, *β*_pri, max_, when *D*_sep_ changes in range of 6 nm<*D*_sep_<18 nm.”

(Please see Page 13, Lines 5-7.)

14. Please specify how Weber number is calculated or given.

Ans: Thank you for your valuable comment. Please see the following statement in the revised manuscript: “In this work, the increment of speed of impacting droplets is 100 m/s, and the corresponding Weber bumber can be calculated based on *We*=*ρD*_0_*V*_0_^2^/*γ*.”

(Please see Page 11, Lines 4-5.)

15. How did the authors consider the influence of contact angle hysteresis on the behavior of impinging nanodrops?

Ans: Thank you for your valuable comment. For the static wetting, the contact angle hysteresis is one of most important parameter if water droplets are deposited on inclined surfaces. For impacting water droplets, the inertia force takes charge of the spreading process, while the released surface energy drives the retracting process. On the other hand, the contact angle hysteresis only takes the second place. The comment is very suitable, the contact angle hysteresis may play a significant role in impacting binary droplets on inclined surfaces, which can provide a guidance for our future work.

16. Though the authors provided a lot of numerical results, there seems to have a lack of comparison between numerical results and experimental/theoretical results.

Ans: Thank you for your valuable comment. Please see the following statement in the revised manuscript: “For impingement of a water droplet with 20 μm upon on superhydrophobic surfaces, Ref. 49 shows that the droplet starts to spread after it interacts with the solid surface, subsequent retraction occurs with a liquid nail pinning on the surface, and ultimately water droplet leaves off from the surface, as shown in **Figure 2a**. Our simulated results of the impacting water droplet with a diameter of 8 nm is illustrated in **Figure 2b**. These snapshots are consistent with what we have observed in experimental tests (see **Fig. 2a**). The tiny deviation may come from the following two reasons. First, the present system adopts a nanoscale droplet with only 8 nm while the size of the impacting droplet in experiment is tens of micrometers. Another one is that the environment of experimental observation is normal pressure and temperature, while MD simulations are performed with a vacuum environment.”

(Please see Page 8, Lines 18-23; see Page 9, Lines 1-4)

**Figure 2.** (a) Experimental results of impacting droplet with 20 μm upon a superhydrophobic solid surface. (b) Sequential images of impingement of a nanodroplet with diameter of 8 nm from MD simulations.

(Please see Page 9.)
