# Supplementary material for: Impingement of systems containing suspending droplets and coming ones at the nanoscale
Source: PLoS One. 2026 Feb 2;21(2):e0340439. doi: 10.1371/journal.pone.0340439 (PMC12863518; doi:10.1371/journal.pone.0340439)
Supplement: Attachment — Submitted filename: Response to Journal and Editor.docx [file pone.0340439.s003.docx]

Journal Requirements:

1. If the reviewer comments include a recommendation to cite specific previously published works, please review and evaluate these publications to determine whether they are relevant and should be cited. There is no requirement to cite these works unless the editor has indicated otherwise.

Ans: Thank you for your valuable comment. We have confirmed that we do not cite works recommended by reviewers.
