# Supplementary material for: Impingement of systems containing suspending droplets and coming ones at the nanoscale
Source: PLoS One. 2026 Feb 2;21(2):e0340439. doi: 10.1371/journal.pone.0340439 (PMC12863518; doi:10.1371/journal.pone.0340439)
Supplement: Attachment — Submitted filename: commetns for PONE-D-25-31635R2 .docx [file pone.0340439.s004.docx]

This manuscript presents a molecular dynamics study on the impingement dynamics of binary nanodroplets with a height difference. The topic is relevant to the micro/nanofluidics community and applications such as inkjet printing. The study systematically investigates the effects of separation distance ($D_{sep}$) and Weber number ($We$) on the primary and secondary spreading, as well as the contact time. The identification of two distinct spreading regimes and the analysis of the prefactor change are interesting findings. While the study is scientifically sound and provides valuable insights, the manuscript requires significant revisions, particularly in language clarity, presentation, and depth of mechanistic explanation, before it can be considered for publication.

1．The manuscript suffers from numerous grammatical errors, awkward phrasing, and repetitive statements, which significantly hinder comprehension. Examples include: "obviously postpone the collision obviously", "recordation of the maximal spreading factor", and "the impingement of multiple droplets is more close to practical applications". A thorough proofreading by a native English speaker or a professional editing service is strongly recommended.

**2．**The transition between sections is sometimes abrupt. Specifically, the "Results and Discussion" section begins directly after a brief mention of model validation in Section 3. A dedicated subsection or a clearer narrative link would improve the flow. The conclusions, while summarizing the findings, could be more impactful by better synthesizing the physical insights and their broader implications.

**3．**The rationale for the scaling law $\beta_{\max}\sim We^{1/2}\mathrm{Re}^{1/5}$needs a more detailed explanation. While it's mentioned that viscous effects are non-negligible at the nanoscale, a clearer connection between the energy balance (including viscous dissipation) and the chosen scaling should be provided. The discussion on how "incomplete-retraction collision" transitions from suppressing to promoting secondary spreading is a key point. However, the explanation, especially regarding the "weakened van der Waals force" and "rigidity", remains somewhat qualitative and speculative. A more quantitative analysis or a clearer physical mechanism would strengthen this central claim. The description of the energy dissipation calculation in Figure 8d is insufficient. The method for calculating the "proportion of energy dissipation" should be explicitly stated in the main text or figure caption.

4.Some terms are used inconsistently, e.g., "secondary spreading" vs. "second spreading". Please standardize the terminology throughout the manuscript. The formatting of variables (e.g., $D_{sep}$ $We$) is inconsistent, sometimes with subscripts and sometimes without. Please ensure all variables are typographically consistent.
