# Supplementary material for: Impingement of systems containing suspending droplets and coming ones at the nanoscale
Source: PLoS One. 2026 Feb 2;21(2):e0340439. doi: 10.1371/journal.pone.0340439 (PMC12863518; doi:10.1371/journal.pone.0340439)
Supplement: Attachment — Submitted filename: Response to Journal and Editor (1).docx [file pone.0340439.s005.docx]

**Response to Editor**

We have carefully reviewed the revisions made by the authors in response to the comments raised in the previous round of review. All the concerns and suggestions put forward earlier have been adequately addressed with thorough and reasonable revisions, which have significantly improved the quality and completeness of the manuscript. We believe the manuscript now meets the academic standards and publication requirements of the journal. We suggest bypassing the "Comments to Authors" section of this round and recommend its acceptance for publication.

**Journal Requirements**

1. If the reviewer comments include a recommendation to cite specific previously published works, please review and evaluate these publications to determine whether they are relevant and should be cited. There is no requirement to cite these works unless the editor has indicated otherwise.

Ans: Thank you for your valuable comment. We have confirmed that we do not cite works recommended by reviewers.

2. Please review your reference list to ensure that it is complete and correct. If you have cited papers that have been retracted, please include the rationale for doing so in the manuscript text, or remove these references and replace them with relevant current references. Any changes to the reference list should be mentioned in the rebuttal letter that accompanies your revised manuscript. If you need to cite a retracted article, indicate the article’s retracted status in the References list and also include a citation and full reference for the retraction notice.

Ans: Thank you for your valuable comment. We have reviewed our reference list to ensure it is complete and correct, and does not cite retracted papers.
